# In-Air Behavioral Audiograms of Two Australian Sea Lions (*Neophoca cinerea*)

**DOI:** 10.3390/ani16152342

**Published:** 2026-08-01

**Authors:** Christine Erbe, Brad McKenzie, Jacob Stek, Lachlan Gill, Elysia Kriek, Malcolm Perry, Benjamin J. Pitcher, Evgenii Sidenko, David Slip, Adrienna van Gogh, Chong Wei

**Affiliations:** 1 Centre for Marine Science and Technology, Curtin University, Bentley, WA 6102, Australia; lachlan.gill@curtin.edu.au (L.G.); m.perry@curtin.edu.au (M.P.); evgeny.sidenko@curtin.edu.au (E.S.); chong.wei@curtin.edu.au (C.W.); 2 Taronga Institute of Science and Learning, Taronga Conservation Society Australia, Mosman, NSW 2088, Australia; bmckenzie@zoo.nsw.gov.au (B.M.); jstek@zoo.nsw.gov.au (J.S.); ekriek@zoo.nsw.gov.au (E.K.); bpitcher@zoo.nsw.gov.au (B.J.P.); dslip@zoo.nsw.gov.au (D.S.); avangogh@zoo.nsw.gov.au (A.v.G.); 3 School of Natural Sciences, Macquarie University, Sydney, NSW 2109, Australia

**Keywords:** Australian sea lion, *Neophoca cinerea*, audiogram, hearing sensitivity, behavioral audiometry

## Abstract

The Australian sea lion (*Neophoca cinerea*) is an endangered species with threats including bycatch, prey depletion, pollution, and habitat loss. Coastal and offshore industrial development causes disturbance—largely mediated by noise. Yet nothing was known about this species’ hearing sensitivity. We present the first in-air audiograms of two adult female Australian sea lions, measured between 100 Hz and 20 kHz via behavioral audiometry. Greatest sensitivity occurred at 3.2 kHz at 13 and 14 dB re 20 µPa in the two individuals. Their 20 dB bandwidth of best sensitivity ranged from 600 Hz to 9 kHz in one and to at least 20 kHz in the other sea lion. While these audiograms were generally comparable to those published for other sea lion species, frequencies of best sensitivity were lower in the Australian sea lions. These results may add to future research on comparative ecology in pinnipeds and inform the management of anthropogenic noise.

## Simple Summary

Hearing is important for Australian sea lions (*Neophoca cinerea*) to mate, rear their young, socialize, find food, and avoid potential threats, but little was previously known about their hearing abilities. We trained two female Australian sea lions at Taronga Zoo, Sydney to participate in aerial hearing tests. Tones of various frequencies and levels were played to the animals, and they indicated when they could hear a tone by pressing a paddle. Both individuals were most sensitive between 600 Hz and 20 kHz. These results will help assess how human-made noise may affect this endangered species.

## 1. Introduction

The Parvorder Pinnipedia comprises the families Odobenidae (walruses), Otariidae (fur seals and sea lions), and Phocidae (true seals). These animals are amphibious, spending part of their time in water (for foraging and, in some species, mating) and part of their time on land or ice (for mating, birthing and pup rearing, molting, and ocean predator evasion) [1,2]. Conservation status differs with species and location. Most are listed as Least Concern, having stable or growing population numbers. However, others are Vulnerable, Threatened, or Endangered. The Caribbean monk seal (*Neomonachus tropicalis*) [3] and the Japanese sea lion (*Zalophus japonicus*) [4] became extinct within the past 70 years. Threats include climate change, prey depletion, habitat degradation, bycatch, and marine pollution [5].

The Australian sea lion (*Neophoca cinerea*) is one of the most endangered otariids with a population size of ~12,000. They occur from southwestern Western Australia to South Australia. The number of breeding colonies and the species’ breeding range are in decline. The species was heavily hunted following European settlement of Australia. Entanglement in fishing gear, illegal killing, pollution, and disease are current causes of mortality [6]. A growing concern are coastal and offshore development (urbanization, shipping and ports, and offshore energy), leading to habitat loss and disturbance [7]—largely mediated by noise.

Hearing is a critical sensory modality for pinnipeds [8,9,10], facilitating communication, navigation, prey detection, environmental awareness, and predator avoidance [11]. However, their amphibious ecology presents unique challenges for hearing in air and under water—two media with greatly different acoustic impedances [12]. Understanding auditory sensitivity in pinnipeds is fundamental to interpreting their behavioral ecology and assessing the potential impacts of anthropogenic noise in both coastal and marine habitats. The complete lack of audiometric information on Australian sea lions presents a significant knowledge gap.

The best studied otariid—with regard to hearing sensitivity—is the California sea lion (*Zalophus californianus*). Audiograms (i.e., curves representing hearing threshold as a function of frequency) have been measured from both males and females in air [13,14,15,16] and under water [15,17,18,19,20]. Audiograms are also available for the Northern fur seal (*Callorhinus ursinus*) [13,21] and the Steller sea lion (*Eumetopias jubatus*) [22,23] in air and under water.

Several audiometric methods exist and they may be grouped into physiological or behavioral [24]. A common physiological technique measures auditory evoked potentials (AEPs), in which electrodes are placed on the animal’s head, an acoustic stimulus (i.e., a sound) is presented, and (neuro-)electric responses are recorded. Thus, AEPs assess the peripheral auditory system, comprising outer, middle, and inner ears, along with auditory nerves. AEPs quantify how the ear collects sound, amplifies/filters it, and converts vibrations into neural signals [25].

Behavioral techniques add one further process—perception. By operant conditioning, the animal is trained to respond to an acoustic stimulus with a certain behavior (e.g., pressing a response paddle) [26,27]. In positive reinforcement, the animal is rewarded for correct responses and merely restationed for the next trial upon incorrect responses. Behavioral audiograms are considered the “gold class” of hearing curves as they assess the full auditory process [24].

This study presents the first behavioral audiogram of the Australian sea lion in air, measured from two individuals at Taronga Zoo, Sydney. These data establish a baseline for future investigations of Australian sea lion auditory ecology and provide essential information for evaluating the potential impacts of noise on this endangered species.

## 2. Materials and Methods

Hearing tests were conducted inside the main entrance room of the former aquarium at Taronga Zoo, Sydney, NSW, Australia, in May and June 2026. This room was close to the holding pools of the animals, which allowed moving individuals in and out easily. The doors and windows of the room were sound-proofed with high-density, 50 mm thick, noise absorbing boards (CSR Martini, Ingleburn, NSW, Australia). An anechoic chamber was constructed inside the room, which consisted of five 2258 mm wide and 2000 mm tall noise control walls (ControlHire, Seven Hills, NSW, Australia) set up in a pentagon. One of the walls was set on wheels, so that it could be rolled open and closed. The roof was made of a layer of PVC noise barrier blankets (ControlHire) and a layer of absorbing boards (CSR Martini).

**Figure 1:**
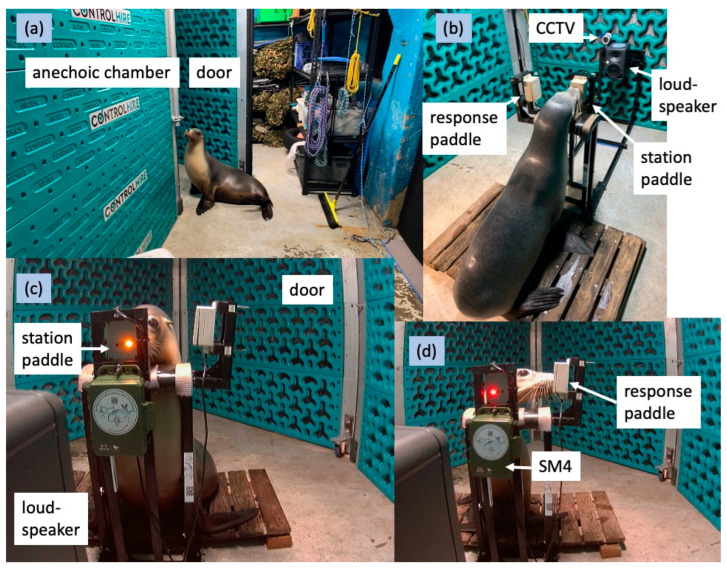
Photos of (**a**) Tarni walking into the anechoic chamber; (**b**) Nala stationing for a hearing test; (**c**) Tarni holding station, view from the CCTV, note the yellow diode indicating the animal is stationed properly; (**d**) Nala pressing the response paddle upon hearing a tone played from the loudspeaker in front of her, view from the CCTV, note the red diode indicating suppression of the response paddle.

The electronic equipment included a Neumann KH 80 DSP Active Studio Monitor (frequency range 60 Hz–21 kHz; Neumann, Berlin, Germany) for sound projection. A closed-circuit television (CCTV) system (DVR8-5580G; Swann Communications, Port Melbourne, VIC, Australia) with a single camera, mounted above the loudspeaker and facing the animal, transmitted live video of the animal’s responses inside the anechoic chamber to the experiment conductor in the adjacent office. A frame was constructed out of modular square tubes of aluminum and polyethylene joiners (Metal Mate; RCR, Cranbourne West, VIC, Australia), which held the loudspeaker, video camera, chin rest, station paddle, and response paddle. The chin rest was mounted 1000 mm in front of the loudspeaker. Two paddles were custom-made: the station paddle at the chin rest in line with the loudspeaker, and the response paddle to the side of the animal’s head. Each paddle was wired to a control box in the adjacent office. A yellow diode lit up when the animal pressed the station paddle, and a red diode lit up when the animal pressed the response paddle. A Song Meter SM4 acoustic recorder (Wildlife Acoustics, Maynard, MA, USA) was mounted below the chin station to record the entire experiment. Sound pressure levels at the SM4 and at the outer ear pinnae of the animals were calibrated with a B&K 2270 Analyzer (ZF-weighted; i.e., unweighted, fast 250 ms integration; Brüel & Kjær, Nærum, Denmark) once the noise absorbing structures and entire experiment had been set up. We took the mean of the levels at the two ear positions. Ambient noise was measured at the ear positions as well, with the B&K 2270, at the time of calibration. During the experiment, the operator controlled which tones were played via a laptop in the adjacent office. The experimental setup inside the anechoic chamber is shown in Figure 1.

Two adult female Australian sea lions were trained to participate in the hearing tests. Nala had been born at the Zoo in January 2009 and was 17 years old. Tarni was wild born, estimated in March 2016. She was found orphaned on the South Australian coast. She was 10 years old at the time of the audiometric experiment. Nala weighed 60 kg, Tarni 90 kg. Tarni was in the late stages of gestation, having been mated in February 2025 (i.e., 16 months prior to data collection; the gestation period in Australian sea lions is 18 months including 4 months of diapause [28,29]). Both Nala and Tarni were involved in shows at the time of the experiment.

During the experiment, the animal positioned itself at the chin rest in front of the speaker and pressed the station paddle with its nose. The yellow diode indicated to the experiment conductor when the animal was stationed properly. Randomly within a 4 s window, a tone was played (signal trial) or not (catch trial). About 30–50% of trials were catch trials, with more catch trials near the emerging hearing threshold. If the animal heard the tone, it pressed the response paddle to its left, otherwise, it held station (go/no-go paradigm). Only the experiment conductor knew what trial was played and observed the animal response on the CCTV screen and control box. In the case of a correct response (i.e., pressing the response paddle when a tone was played and holding station for 4 s on catch trials), the conductor shouted ‘yes’ into the chamber, upon which a trainer inside the chamber blew a whistle, upon which the animal turned towards the trainer for reward (a random piece of fish from the food bucket allocated to this session). In the case of a false response (i.e., breaking from the station paddle prior to tone transmission, breaking on a catch trial, or missing a tone), the conductor shouted ‘no’ into the chamber, no whistle sounded, no reward was given, and the animal was restationed. Pressing the response paddle prior to tone transmission (pre-tone response) or on a catch trial was counted as a false alarm. The false alarm rate was calculated as the number of false alarms divided by the sum of pre-tone responses and catch trials.

Only one tone frequency was played during a session. Mostly, two sessions (1–3) were run per day, 3–4 days per week. The very first tones were aimed at 40 dB above the California sea lion threshold [14,16]; in subsequent sessions, we started at 20–30 dB above the emerging Australian sea lion threshold. The sound pressure level of the tone was decreased in 6 dB steps until the animal stopped responding; it was then increased again in 6 dB steps until the animal indicated that it heard it again, after which it was decreased again, and eventually increased again (staircase procedure). The hearing threshold was computed as the mean of the reversal levels. Tone frequencies ranged from 100 Hz to 20 kHz. Tones were pure sinusoids, 1 s in duration, including 20 ms for ramp-up and 20 ms for fade-out. Each frequency was tested in three sessions and the lowest threshold reported.

## 3. Results

**Figure 2:**
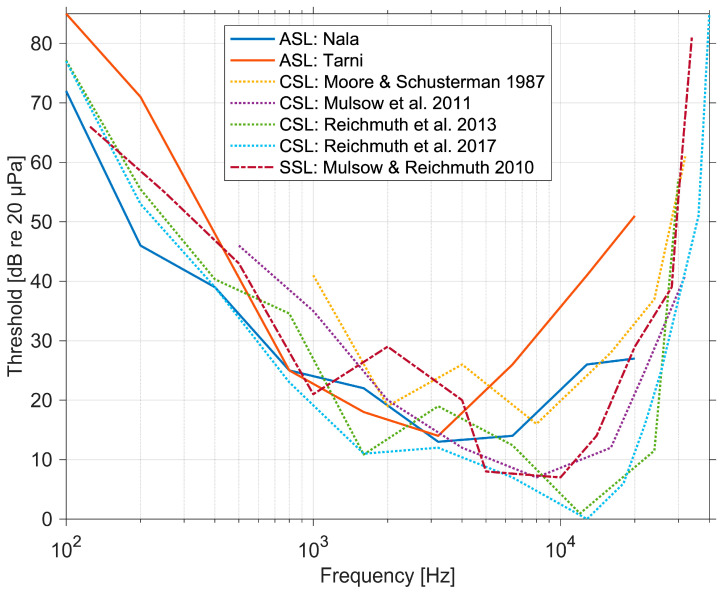
In-air audiograms of the two adult female Australian sea lions (ASL) measured at Taronga Zoo, compared to those published from California sea lions (CSL) [13,14,15,16] and a Steller sea lion (SSL) [23].

The in-air audiograms of Nala and Tarni are shown in Figure 2, together with those published of other sea lions. The thresholds are also given in Table 1, together with ambient noise measurements. Both individuals were most sensitive at 3.2 kHz, with thresholds of 13 and 14 dB re 20 µPa. Nala’s frequency band of best sensitivity (measured at 20 dB above the lowest threshold) extended from ~600 Hz to probably just above 20 kHz. Tarni’s 20 dB bandwidth of best sensitivity ranged from ~600 Hz to 9 kHz. While hearing thresholds for the two individuals were similar at 800–3200 Hz, Tarni appeared less sensitive than Nala at lower and higher frequencies. Of note, Tarni also had a greater false alarm rate than Nala. At the beginning of a session, when tone levels were high, neither animal gave false alarms. As the tone level decreased, the false alarm rates increased—at all frequencies. Across the experiment, Nala’s false alarm rate was ~20% and Tarni’s ~35%.

## 4. Discussion

We presented the first in-air audiograms of Australian sea lions between 100 Hz and 20 kHz. We were unable to project tones >20 kHz (due to equipment limitations) and so are missing the high-frequency cut-off of the audiogram, where the hearing threshold is expected to rise steeply with frequency [8].

One of our animals (Tarni) was less sensitive than the other (Nala) at the lowest and highest frequencies tested. Both were adult females; Tarni was 7 years younger than Nala. Given that Tarni was a wild-born, stranded orphan, her early life history was unknown. She might have suffered some hearing impairment, as could happen from strong noise exposure or ear infection [30,31,32]. Neither animal had a record of ototoxic medication while under zoo care. Tarni was in the late months of gestation at the time of the experiment, which might have affected her ‘focus’. Her response bias was more liberal than Nala’s, as indicated by Tarni’s greater false alarm rate. However, a greater false alarm rate, and thus, a more liberal bias, tend to yield lower thresholds, not higher [14].

Both Australian sea lion audiograms compared well with those of other sea lion species (i.e., California and Steller) below 3.2–6.4 kHz. Otariid species have similar acoustic ecologies. The sounds they produce (such as barks, roars, and growls) have comparable spectro-temporal features with peak energy below 4 kHz (see overview in [11]). In all otariids, these sounds function in territorial competition, mating, and pup rearing. Consequently, hearing sensitivity is expected to be similar in these species, with the lowest threshold of ~10 dB re 20 µPa in the kHz range [14,33]. For noise regulation and conservation management, otariid species have hence been joined into a functional hearing group [34,35].

Nonetheless, the frequencies of best sensitivity were lower in the two Australian sea lions than in other sea lion species. Both Australian sea lion audiograms missed the high-frequency notch (at 12.8 kHz) reported for some California sea lions [15,16]. The only other otariid audiograms published are from Northern fur seals, with best sensitivity at 5 kHz [21] (i.e., more like our Australian sea lions) and 12.8 kHz [13] (i.e., more like California sea lions).

There are several potential reasons for the discrepancies at high frequencies. We cannot exclude the possibility of at least the older of our two females (Nala, 17 years) to have age-related loss of high-frequency sensitivity (presbycusis [36]). Moreover, our thresholds carry less accuracy than others published, given the differences in step size (6 dB in our study, versus 2–4 dB in others [16]). Differences at high frequencies may also partly be explained by calibration uncertainty. Levels measured at the two ear positions differed by maximally 1 dB at the greatest wavelengths (lowest frequencies) and increased to 7 dB at 20 kHz. This difference could be reduced in future study by mapping out the sound field surrounding the ears and adjusting speaker position to minimize the left-right difference (as demonstrated by Reichmuth et al. [16]).

We investigated whether ambient noise could have masked lower-level tones. We measured ambient noise inside the anechoic chamber at the time of tone calibration, but were unable to track any variability over time, which adds to the uncertainty. Even though we could not accurately measure the ambient noise at the highest frequencies inside the anechoic chamber due to the sensitivity limit of our spectrum analyzer, the levels were still low enough so as not to mask the test tones.

Critical ratios measure how far above a broadband ambient noise the tone level has to be for it not to be masked by the noise [37]. Critical ratios of otariids are ~25 dB at 6.4 kHz, ~28 dB at 12.8 kHz, and ~31 dB at 20 kHz [8,38,39]. Our ambient noise at these frequencies was less than (or equal to) the sensitivity limit of the spectrum analyzer: −20, −23, and −25 dB re (20 µPa)^2^/Hz, respectively. Adding the critical ratios at these frequencies yields sound pressure levels of 5, 5, and 6 dB re 20 µPa—quieter tones are expected to be masked. However, the lowest level to which our animals responded was higher (i.e., 14 dB re 20 µPa) and hence not expected to have been masked.

There are, of course, no critical ratio measurements from Australian sea lions, and this is a need for future research. Critical ratios would allow us to estimate how much of the signals important to sea lion behavior and survival are masked by anthropogenic noise. Critical ratios may be measured in behavioral experiments, similar to those conducted here for audiograms. Background noise would need to be played at controlled spectral levels, and the hearing threshold of pure tones measured in this noise. Masking may also be measured with complex signals (such as sea lion barks) in realistic noise (such as airborne construction or ship noise) [40]. Finally, given that sea lions spend much of their time under water and given that hearing is influenced by physical and physiological mechanisms that differ in air and water, hearing and masking should be tested under water in future.

## 5. Conclusions

We presented the first measurements of in-air hearing sensitivity in Australian sea lions. Individual differences at the lowest and highest frequencies remain unexplained and additional individuals of varying demography and life history should be tested. Hearing ranges and sensitivities were broadly comparable to those reported for other otariid species; however, the Australian sea lions were less sensitive above 6.4 kHz. Ambient noise levels were unlikely to have masked the thresholds, supporting the reliability of our results for these two individuals. These audiograms may inform the management of terrestrial anthropogenic noise. Hearing tests under water are a need for future research and conservation management.

## Figures and Tables

**Table 1:** Frequency of the pure tones tested, hearing thresholds of the two Australian sea lions, ambient noise measured as 1/3 octave band levels (1/3 OBL), and ambient noise power spectral density (PSD) computed from the 1/3 OBLs under the assumption of a flat spectrum within each 1/3 octave band.

Frequency [Hz]	Nala’s Threshold [dB re 20 µPa]	Tarni’s Threshold [dB re 20 µPa]	Ambient Noise 1/3 OBL [dB re 20 µPa]	Ambient Noise PSD [dB re (20 µPa)^2^/Hz]
100	72	85	34	20
200	46	71	26	9
400	39	48	18	−2
800	25	25	15	−8
1600	22	18	14	−12
3200	13	14	11	−18
6400	14	26	12	−20
12,800	26	41	12	−23
20,000	27	51	12	−25

## Data Availability

The original contributions presented in this study are included in the article. Further inquiries can be directed to the corresponding author.
